# Computational comparison of different plating strategies in medial open-wedge high tibial osteotomy with lateral hinge fractures

**DOI:** 10.1186/s13018-020-01922-0

**Published:** 2020-09-14

**Authors:** Yen-Nien Chen, Chang-Han Chuang, Tai-Hua Yang, Chih-Wei Chang, Chun-Ting Li, Chia-Jung Chang, Chih-Han Chang

**Affiliations:** 1 Department of Physical Therapy, Asia University, 500, Lioufeng Rd., Wufeng, Taichung, 41354 Taiwan; 2 Department of Orthopedics, Show Chwan Memorial Hospital, Changhua City, Taiwan; 3 Department of Biomedical Engineering, National Cheng Kung University, Tainan, Taiwan; 4 Department of Orthopedics, National Cheng Kung University Hospital, College of Medicine, National Cheng Kung University, Tainan, Taiwan; 5 Skeleton Materials and Bio-compatibility Core Lab, Research Center of Clinical Medicine, National Cheng Kung University Hospital, College of Medicine, National Cheng Kung University, Tainan, Taiwan; 6 Medical Device Innovation Center, National Cheng Kung University, Tainan, Taiwan; 7 Department of Orthopedics, College of Medicine, National Cheng Kung University, Tainan, Taiwan; 8 Institute of Geriatric Welfare Technology & Science, Mackay Medical College, New Taipei City, Taiwan

**Keywords:** High tibial osteotomy, Lateral hinge fracture, Plating strategy, Finite element simulation

## Abstract

**Background:**

Lateral hinge fracture (LHF) is associated with nonunion and plate breakage in high tibial osteotomy (HTO). Mechanical studies investigating fixation strategies for LHFs to restore stability and avoid plate breakage are absent. This study used computer simulation to compare mechanical stabilities in HTO for different LHFs fixed with medial and bilateral locking plates.

**Methods:**

A finite element knee model was created with HTO and three types of LHF, namely T1, T2, and T3 fractures, based on the Takeuchi classification. Either medial plating or bilateral plating was used to fix the HTO with LHFs. Furthermore, the significance of the locking screw at the combi hole (D-hole) of the medial TomoFix plate was evaluated.

**Results:**

The osteotomy gap shortening distance increased from 0.53 to 0.76, 0.79, and 0.72 mm after T1, T2, and T3 LHFs, respectively, with medial plating only. Bilateral plating could efficiently restore stability and maintain the osteotomy gap. Furthermore, using the D-hole screw reduced the peak stress on the medial plate by 28.7% (from 495 to 353 MPa), 26.6% (from 470 to 345 MPa), and 32.6% (from 454 to 306 MPa) in T1, T2, and T3 LHFs, respectively.

**Conclusion:**

Bilateral plating is a recommended strategy to restore HTO stability in LHFs. Furthermore, using a D-hole locking screw is strongly recommended to reduce the stress on the medial plate for lowering plate breakage risk.

## Introduction

High tibial osteotomy (HTO) with a locking plate is a well-established surgical approach to adjust the mechanical axis of the low extremity with medial compartmental knee osteoarthritis (KOA) and restore the joint space of the medial knee of the KOA [1–3]. In recent years, HTO has gained popularity because of its satisfactory clinical outcome, particularly in patients aged 40–60 years [4–6]. Furthermore, HTO slows down the KOA process, and approximately 90% of patients could resume their original work and sports within 1 year of HTO [7]. To ensure the bone healing of the medial open wedge after HTO, stable fixation is required, and recently, the locking plate has been shown to provide excellent stability for early rehabilitation exercise [8, 9].

Although HTO showed satisfactory clinical outcomes, complications such as the need for symptomatic hardware removal, deep wound infection, hardware failure with correction loss, nonunion, early conversion to arthroplasty, displaced (> 2 mm) and undisplaced (< 2 mm) lateral hinge fracture (LHF), delayed wound healing, and undisplaced lateral tibia plateau fracture are common [10]. Other studies have reported an LHF incidence of 19.8–41.2% after HTO [7, 11–13]. Furthermore, LHF is highly related to nonunion and plate breakage [14].

Many studies have investigated LHF incidence after HTO and complications following LHFs. The biomechanical effect of the LHFs on the HTO with medial opening wedge and the bone plate was also revealed [15]. In the study, the stress of the medial plate was increased with the LHFs. However, no mechanical study has investigated the fixation strategies of HTO for LHFs to restore stability and avoid plate breakage. Bilateral plating was demonstrated to re-correct the loss and nonunion after HTO [16], whereas mechanical stability was not investigated. Furthermore, only one type of LHF was involved in the study [16]; the other types of LHFs were not investigated.

The aim of this study was to compare the mechanical stability, including gap shortening at the osteotomy site, the displacement of the proximal tibial fragment, and the stress on the metallic bone plates, in HTO with various LHFs, and medial and bilateral plates by using the finite element (FE) method. The FE method is a numerical tool to obtain mechanical responses, including internal stress and deformation, of the whole model [17, 18]. Additionally, the FE method excludes the variation between the samples in the study.

## Methods

A reliable FE HTO model, which was validated and used to compare HTO stability with various screw configurations in a previous study [19], was modified to conduct HTO with LHFs and following medial and bilateral plate fixation.

### Solid model

**Fig. 1:**
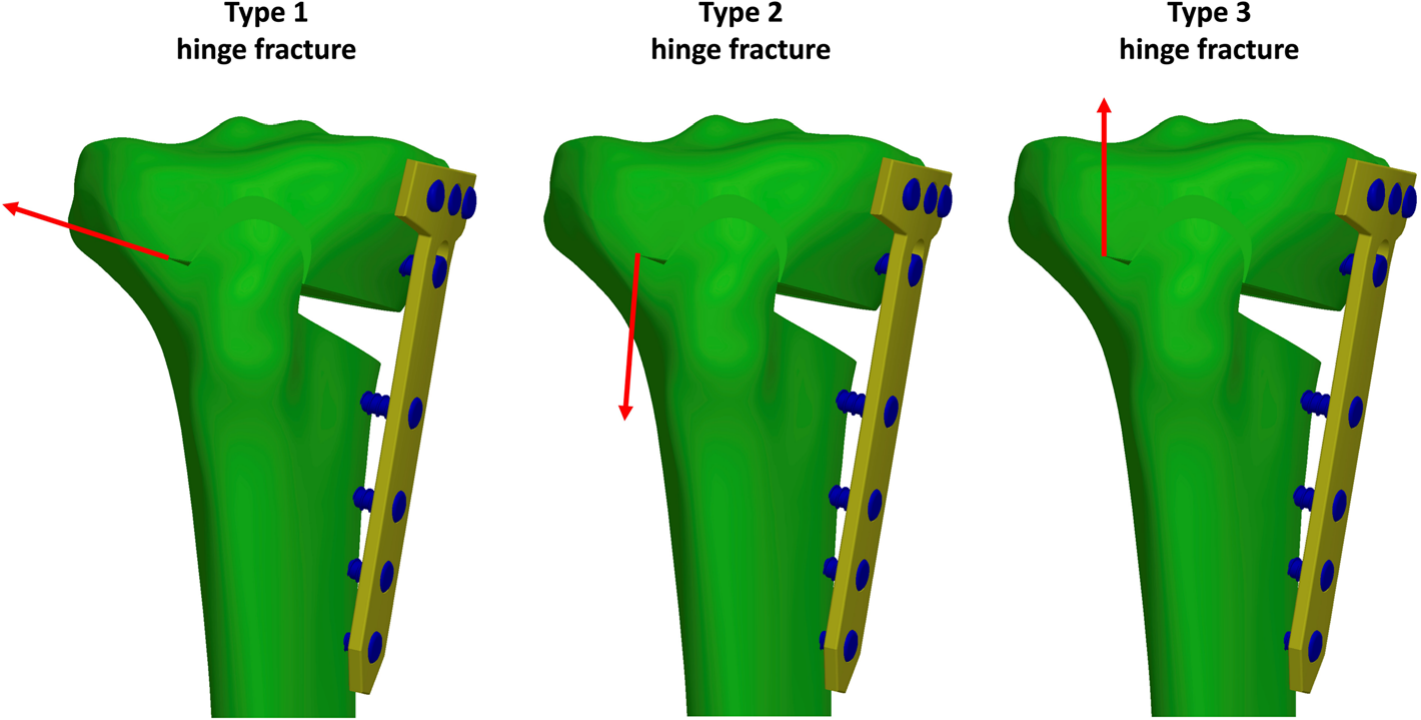
Types of LHFs used in this study

**Fig. 2:**
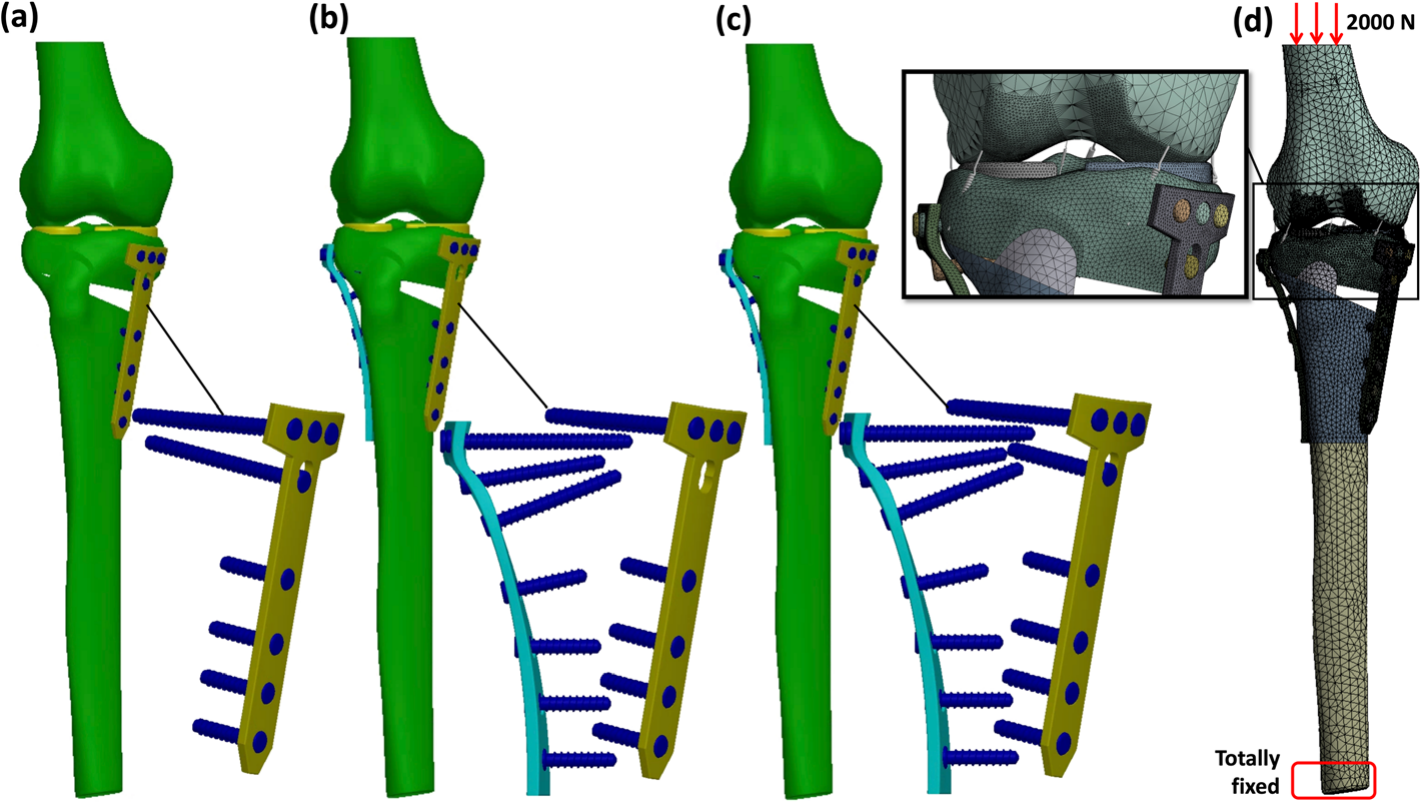
Fixation strategies and bonudary condition used in this study, including **a** MP, **b** BP without D-hole screw, **c** BP, and **d** boundary condition

The HTO model, with medial TomoFix plate and without LHF, used in a previous study was modified according to various hinge fractures. The angle of the medial open wedge and the width of the lateral hinge were set to 12° and 10 mm, respectively. The width of the lateral hinge refers to the distance from the apex of the wedge to the lateral edge of the tibia. In this study, three types of LHF fractures, namely type 1 (T1), type 2 (T2), and type 3 (T3), were created according to the Takeuchi classification [20]. T1 fracture referred to a crack, parallel to the osteotomy line of the open wedge, in the open wedge extending to the proximal site of the tibiofibular joint. T2 fracture referred to a crack in the open wedge extending downwardly to the distal site of the tibiofibular joint. T3 fracture was a lateral plateau fracture, which referred to a crack upwardly to the lateral plateau of the proximal tibia (Fig. 1). The fractures were created with virtual planes using the CAD software SOLIDWORKS 2019 (Dassault Systemes SolidWorks Corp., Waltham, MA, USA). Hence, the fracture surfaces were simplified as planes, and no gap existed at the fracture sites. The micro-geometry of the fractured surface was not considered. Then, different fixation strategies were used to stabilize each fracture type (Fig. 2), including medial plating (MP) with the original TomoFix plate (TomoFix, DePuy Synthes, Oberdorf, Switzerland), bilateral plating (BP) with medial TomoFix plate and proximal lateral tibia locking plate (LCP, proximal tibia plate, DePuy Synthes, Oberdorf, Switzerland), and BP without the locking screw (BPWDS) at the proximal combi hole (D-Hole) of the TomoFix plate. The length and width of the TomoFix plate were 115 and 16 mm, respectively. The thickness of both medial TomoFix and lateral locking plates was set to 3 mm. The lengths of the screw at the proximal tibia (including the D-hole screw) and tibia shift in the MP were 56 and 26 mm, respectively. However, the length of the D-hole screw in the BP group was reduced to 38 mm to dodge the proximal screw of the lateral locking plate. The length of the proximal screw for the lateral locking plate was 56 mm (top row) and 44 mm (second and third row from the top) (Fig. 2b, c). The screw length for the lateral locking plate at the tibia shift was 26 mm. The diameter of all screws was set to 5 mm.

### FE model

The models were imported into the ANSYS Workbench 2019 R3 (Swanson Analysis Systems, Inc., Houston, PA, USA) for the following mesh and simulation. Quadratic tetrahedral element (solid 187) was used to mesh hole the model with commend “free mesh.” The local element sizes, including screws, plates, cartilage, and the surfaces of the bone in contact with the screws, were refined with commend “sizing.” Totally, 737,119 elements and 1,173,791 nodes were used to mesh the MP model. The same settings were used to mesh the other two models. Four ligaments (Table 1), namely medial collateral ligament, lateral collateral ligament, anterior cruciate ligament, and posterior cruciate ligament (Fig. 2d), around the knee joint were reconstructed with tension-only spring in the ANSYS Workbench. The locations of the springs were defined based on the anatomy. The contact behavior between the screw and plates was set to bond to simulate the locking mechanism of the locking plate and locking screw. The contact behavior between the screws and the surrounding bone was set to frictional surface-to-surface contact behavior. Furthermore, the bone-to-bone contact behavior at the fracture site and the osteotomy site was set to the frictional surface-to-surface contact. The frictional coefficients of the bone-to-bone and bone-to-metal were defined as 0.45 and 0.3, respectively, based on the literature [21]. The contact behavior between the bone and meniscus of the knee joint was set to frictionless.

**Table 1:** Material properties of the springs used in this study to simulate ligaments

Ligament	Numbers of spring	Stiffness (N/mm)	Cross-section area (mm^2^)	Elastic modulus (MPa)
Medial collateral ligament	2	10.6	1.54	345
Lateral collateral ligament	2	10.6	1.54	345
Anterior cruciate ligament	1	17.5	1.29	345
Posterior cruciate ligament	1	20.6	1.92	345

### Material properties and boundary condition

The material property of the locking plate ad screws was set to titanium, and the elastic modulus and Poisson’s ratio were defined according to the engineering database in the ANSYS Workbench. The material properties of the cortical bone, cancellous bone, and meniscus were defined based on the literature [22–24] (Table 2). A 2000-N vertical downward force (approximately 2.5 times the body weight of an 80-kg subject) was applied on the upper surface of the distal femur to simulate the maximum force on the knee while walking [25]. Only the motion in the vertical direction was allowed for the distal femur. The distal surface of the tibia was totally fixed (Fig. 2d).

**Table 2:** Material properties used in this study

	Elastic modulus (MPa)	Poisson ratio
Cortical bone	12 000	0.3
Cancellous bone	430	0.3
Meniscus	100	0.1
Titanium alloy	96 000	0.36

### Criteria

The maximum displacement of the proximal osteotomy fragment and shortening of the osteotomy gap were adopted as indexes to compare HTO stability with LHFs and fixation approaches. The equivalent stress (also called von Mises stress) on plates was used as an index to evaluate the breakage risk. Furthermore, the force on nodes at the transverse plane of the medial plate below the D-hole was calculated to compare the loading on the medial plate with and without LHFs.

## Result

### Stability

**Fig. 3:**
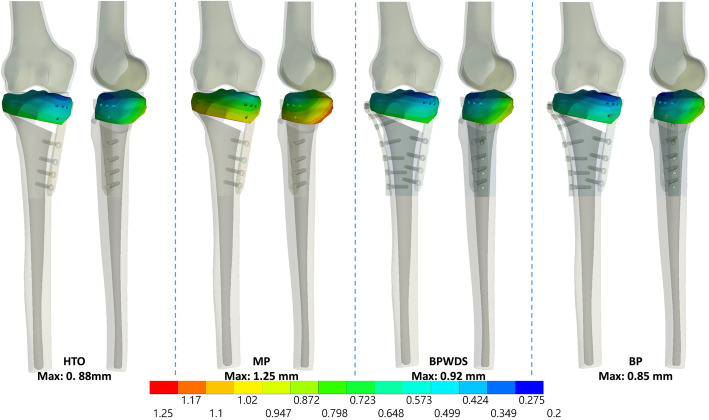
Total displacement of the proximal tibia fragment in HTO and T1 LHF

**Fig. 4:**
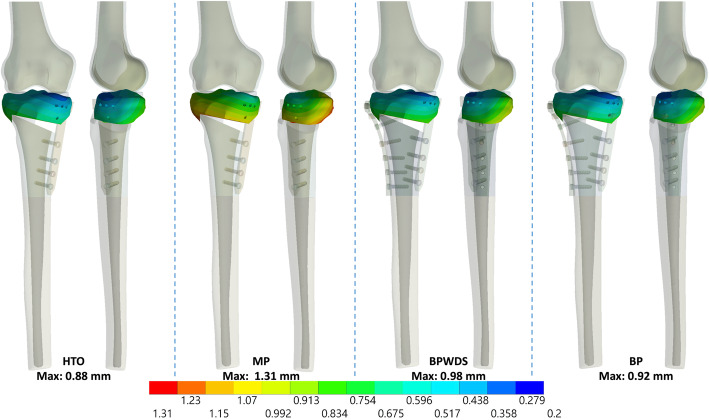
Total displacement of the proximal tibia fragment in HTO and T2 LHF

**Fig. 5:**
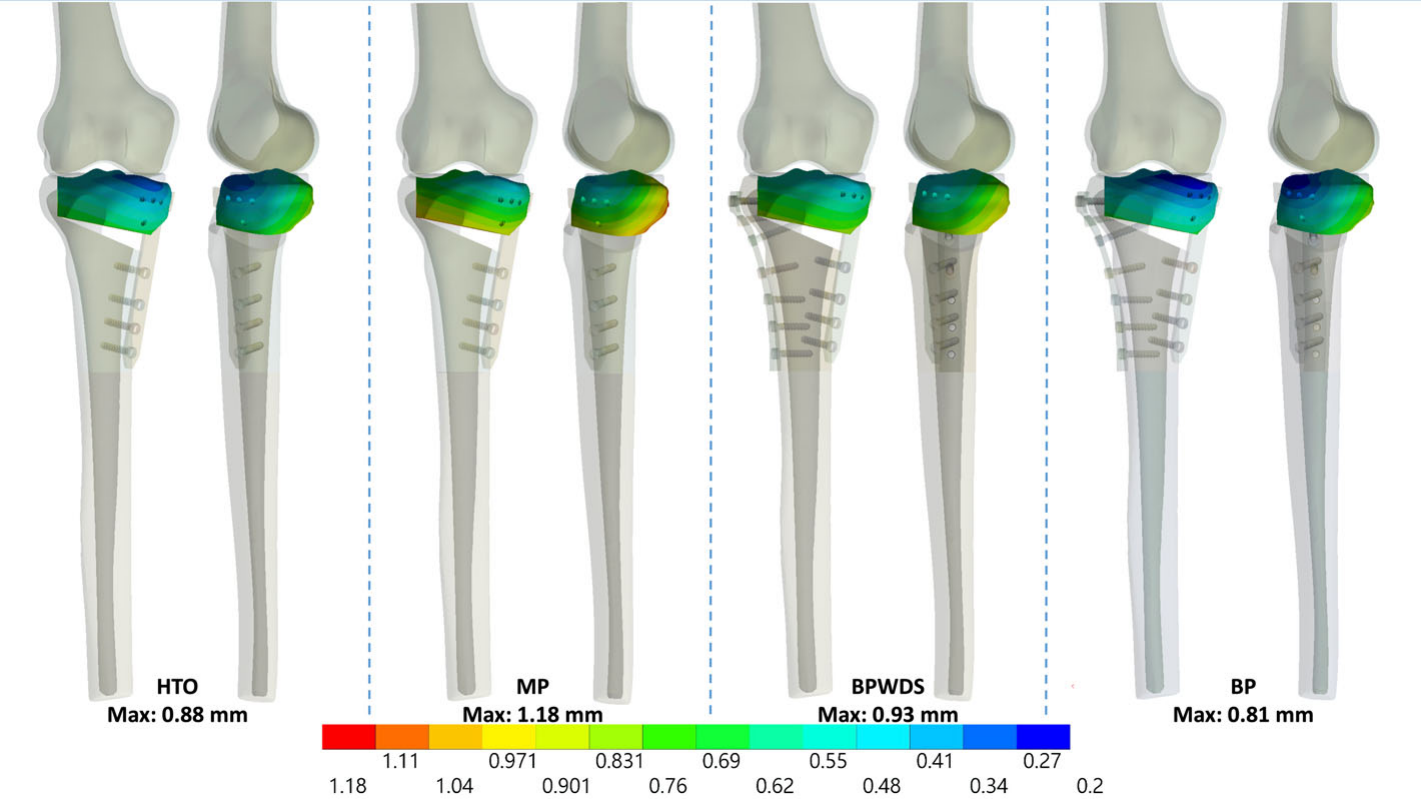
Total displacement of the proximal tibia fragment in HTO and T3 LHF

**Fig. 6:**
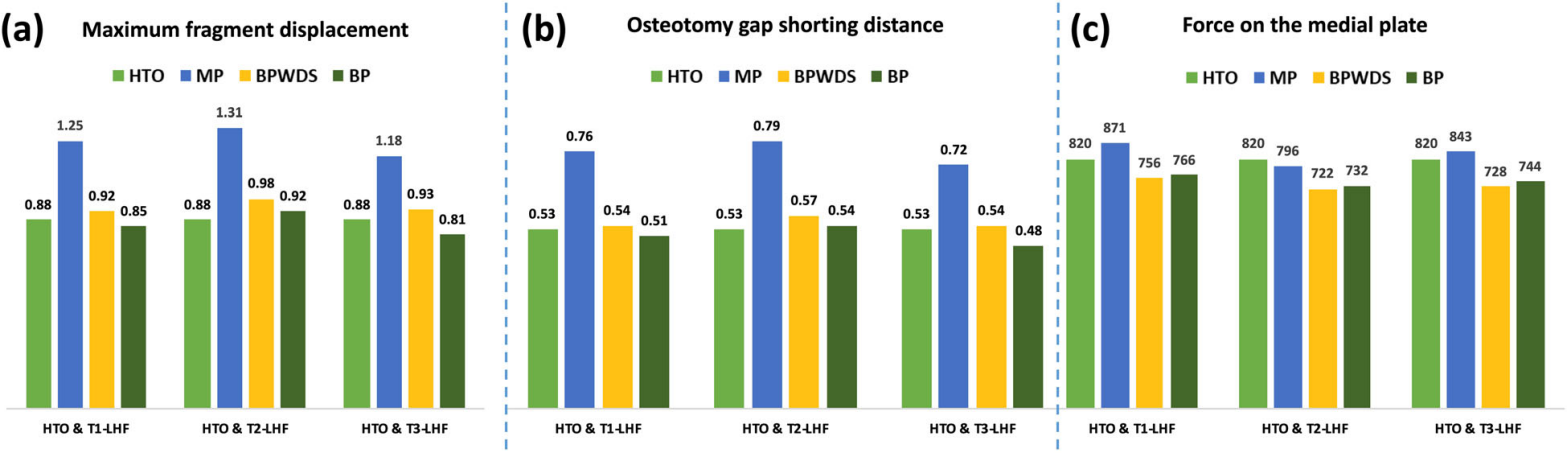
**a** Maximum displacement of the proximal tibia fragment. **b** Gap shortening at the osteotomy site. **c** Total force on the medial plate

Results indicated that LHFs substantially reduced HTO stability when only the medial plate was used, whereas the stability was restored with the addition of the lateral plate, irrespective of the screw used at the D-hole locking (Figs. 3, 4, and 5). The maximum displacement of the proximal tibia fragment with only the MP increased by 42% (from 0.88 to 1.25 mm), 48.9% (from 0.88 to 1.31 mm), and 34.1% (from 0.88 to 1.18 mm) after T1, T2, and T3 LHFs, respectively (Fig. 6a). Additionally, the osteotomy gap shortening distance increased by 43.4% (from 0.53 to 0.76 mm), 49% (from 0.53 to 0.79 mm), and 35.8% (from 0.53 to 0.72 mm), respectively, after T1, T2, and T3 LHFs (Fig. 6). The BP could restore the stability through the reduction in fragment displacement by 32% (from 1.25 to 0.85 mm), 29.8% (from 1.31 to 0.92 mm), and 55.2% (from 1.81 to 0.81 mm) in T1, T2, and T3 LHFs, respectively (Fig. 6a). Furthermore, the BP could maintain the osteotomy gap. The gap shortening distance in HTO without LHFs was 0.53 mm; after LHFs with BP, the gap shorting distances were 0.51, 0.54, and 0.48 mm in T1, T2, and T3 LHFs, respectively (Fig. 6b). Moreover, using the locking screw at the D-hole (BP) reduced the fragment displacement compared with that without the screw (BPWDS).

### Loading on the medial plate

Loading on the medial plate increased slightly after T1 and T3 LHFs but decreased slightly after T2 LHF when only the medial plate was used. However, the loading on the medial plate decreased after adding the lateral plate in LHFs. Using the D-hole screw at the medial plate did not affect loading on the medial plate. The loading on the medial plate in HTO was 820 N, which increased by 51 N and 23 N but decreased by 24 N after T1, T3, and T2 LHFs, respectively (Fig. 6c).

### Stress on the plates

**Fig. 7:**
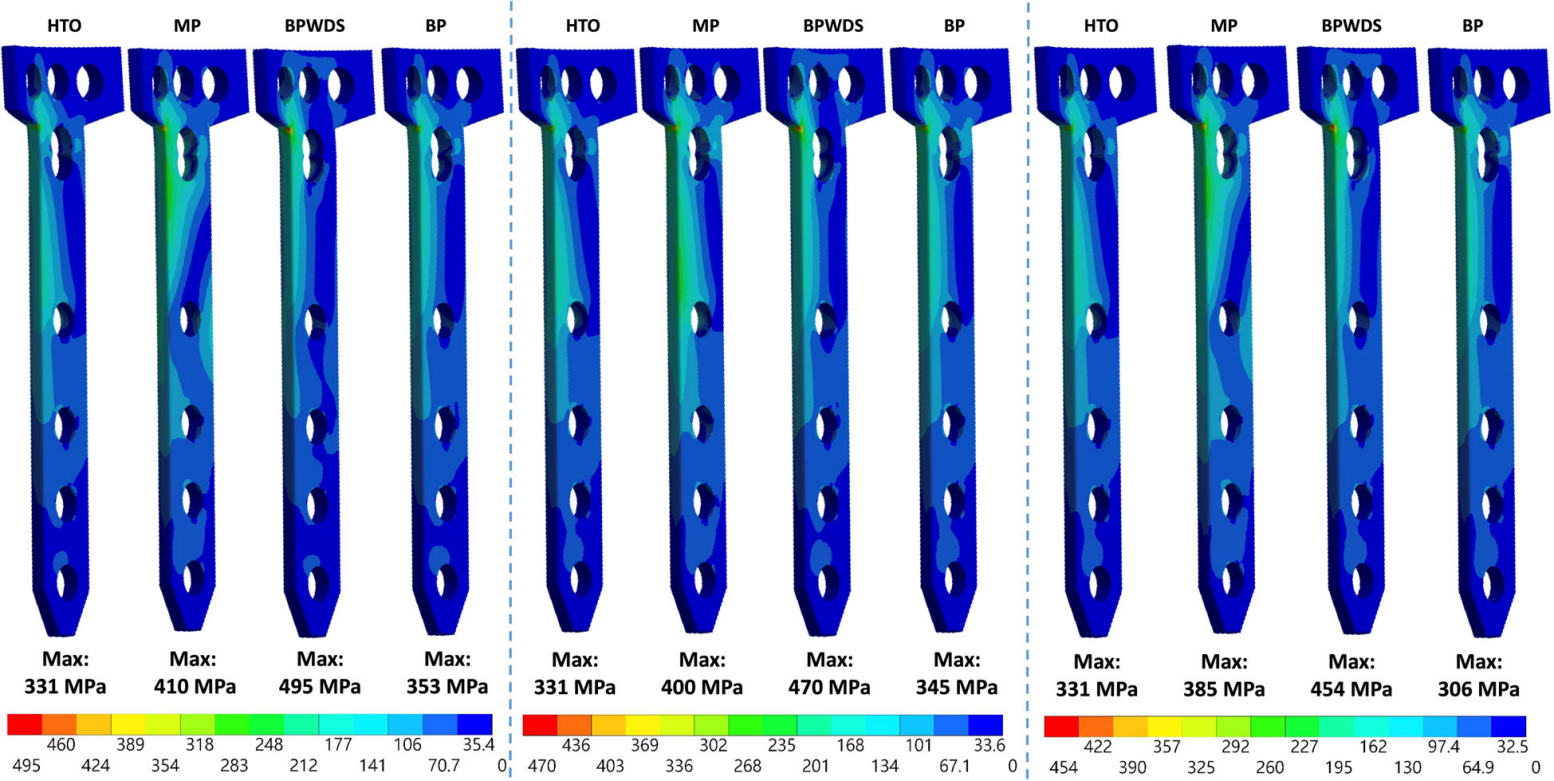
Stress distribution on the medial plate

**Fig. 8:**
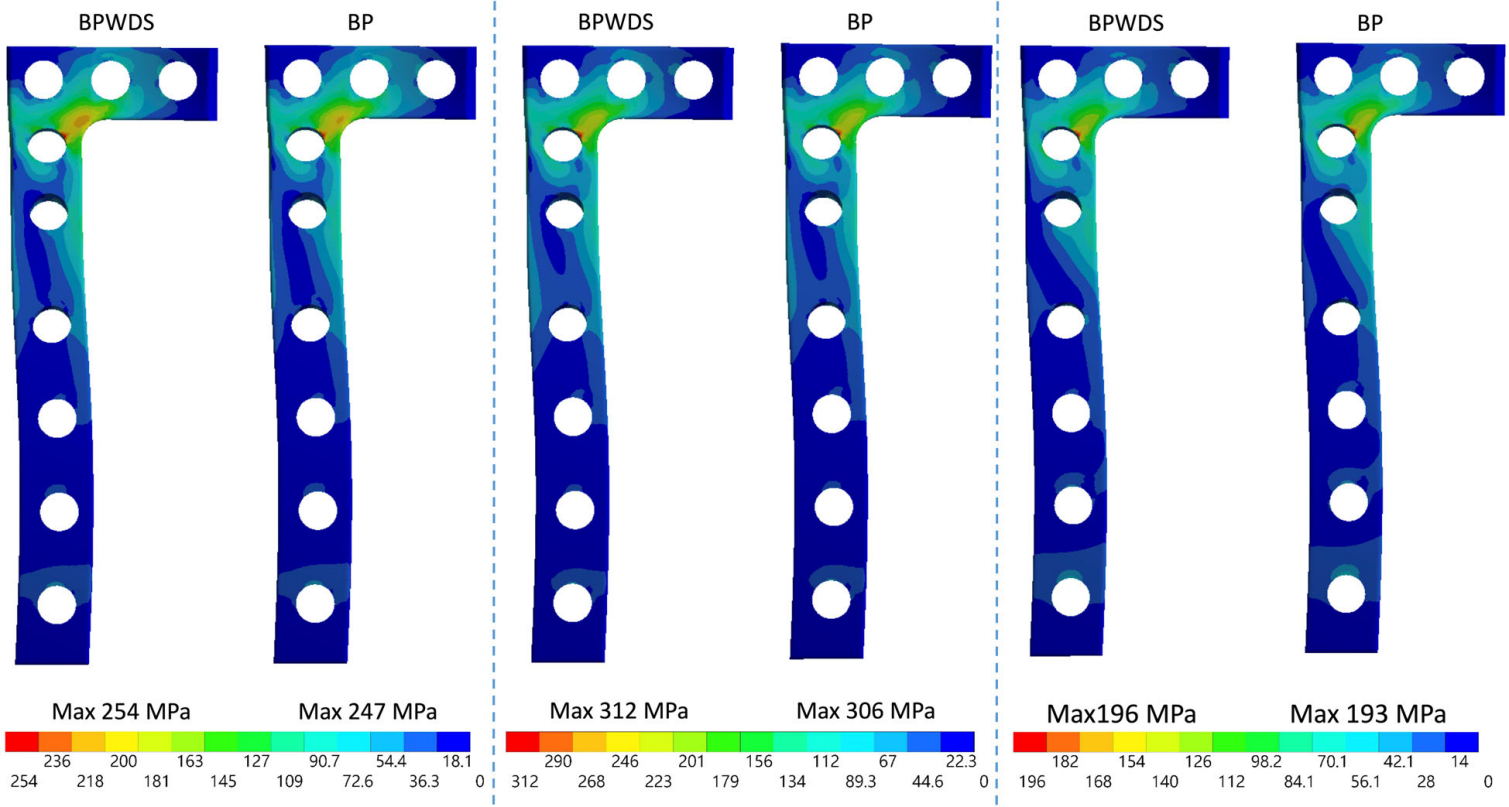
Stress distribution on the lateral plate

The peak equivalent stress on the medial plate clearly increased after LHF. The peak stress on the medial plate increased by 20.8% (from 331 to 400 MPa), 23.9% (from 331 to 410 MPa), and 16.3% (from 331 to 385 MPa) (Fig. 7). Using the lateral plate plus the D-hole screw (BP group) could efficiently reduce the peak stress on the medial plate after LHFs. Moreover, the peak stress on the medial plate increased after LHFs without the D-hole screw although the lateral plate was added. However, using the D-hole screw did not alter the stress on the lateral plate (Fig. 8).

## Discussion

Using HTO for medial compartmental KOA is currently popular because the locking plate and screws can provide excellent stability immediately after osteotomy, enabling the patient to start rehabilitation programs earlier than with the use of the traditional compression plate. Scholars have shown that MP with only the TomoFix locking plate stabilizes HTO with a medial open wedge (without LHF) and provides enough stability for bone healing [26–28]. However, when the lateral hinge is fractured, MP only does not provide adequate stability for bone healing at the osteotomy site and at the fractured lateral hinge. Furthermore, LHFs increased the stress on the medial plate and plate breakage risk. Stability decreases while plate stress increases because the continuous bony structure of the lateral hinge is broken. In this situation, the original medial plate (which is at a distance from the fractured lateral hinge) only was insufficient to stabilize the osteotomized and fractured HTO. Hence, additional fixation, such as lateral plating, is needed for HTO after LHFs.

Mechanically, the medial open wedge is the weakest site of the HTO structure, irrespective of the presence of LHFs. Because no bone medially supports the proximal tibial fragment, a strong support, such as the locking plate, is necessary to substitute the medial bone function. Although the fractured bone at the lateral hinge was able to sustain partial loading, the compressive force, the fractured bone was unable to resist the developed shear or tensile force during the compressive loading in the present simulation. Furthermore, LHFs were at a distance from the MP, and the fixation effect of the medial plate was minimal for LHFs. In such a situation, placing a lateral plate directly at the fractured lateral hinge could efficiently stabilize the tibia with HTO and LHFs.

Plate breakage near the D-hole was highly related to LHFs in a previous study [14], and the result was in accordance with the present FE results. A relative high-stress area appeared near the outside corner and the D-hole of the medial TomoFix plate in HTO, particularly with LHFs in the present study. In the previous FE study [15], the stress of the plate was also increased, particularly in the T2 and T3 LHFs. The high stress after LHFs can create a crack and then extend to the screw hole after cyclic loading during postsurgical walking without bone healing. In the present simulation, BP with the D-hole screw proved to reduce the stress on the medial plate; hence, this approach is recommended in HTO for LHFs.

The locking screw at the D-hole of the medial TomoFix plate is crucial for reducing stress on the plate in BP. In the present simulation, although the stability of the BPWDS was close to that in the BP group, the equivalent stress on the medial plate in BPWDS was much higher than that in the BP group. Furthermore, the equivalent stress on the plate in the BPWDS group was even higher than that in the MP group. The results of the stress indicated the contribution of the D-hole screw to reduce the plate stress. Although the difference in the total loading of the medial plate with and without the screw at the D-hole in bilateral plating was minor, the difference in stress was large. This is because the D-hole screw shunts the force and alerts the total moment developed by the applied loading on the medial plate. Finally, the stress on the plate was lower with the D-hole screw than that without the D-hole screw in BP. In clinical practice, the surgeon may encounter challenges while inserting the D-hole screw. However, the D-hole screw is indispensable for HTO in BP, and the surgeon must prioritize inserting the D-hole screw during HTO for LHFs in BP.

The major concern with BP on the HTO for LHFs is the stress-shielding effect, which leads to a stiff structure during bone healing, leading to callus formation. Recently, the far cortical locking screw and plate have been proposed to reduce structural stiffness and to increase the micromotion of the fracture gap in HTO [29]. In our previous study, the far cortical locking screw reduced the stiffness through an increase in the motion between fragments while increasing the stress on the implant in HTO. Hence, using a far cortical locking screw could be considered a potential strategy for reducing the structural stiffness of the HTO for LHFs by using BP.

T3 LHF is regarded as the worst case among LHFs because the fracture affects the articular surface of the knee joint. Although in the present study the mechanical stability in T3 was better than that in T1 and T2, this does not indicate that T3 LHF is less serious than T1 and T2 LHFs because the deformation of the knee joint surface was not considered in the present simulation. T3 LHF should be treated carefully because the consistency of the articular surface is crucial in the management of an intra-articular fracture. The result of the present mechanical stability is not equal to the final result of bone healing and the functional outcome.

This study has some limitations. First, only the maximum compressive loading conditions in walking were considered. The effect of fixation approaches in the swine phase and tensile loading was not considered. Second, the fibula was not simulated, the trabecular structure of the bone and the micro-geometry of the fractured bone were not considered, and the material properties were simplified as linear elastic, isotropic, and homogeneous. Third, the ligaments around the knee joint were simplified as one-dimensional tension-only springs. Fourth, the original bony structure used was an intact knee without KOA, and only the bone volume of the medial open wedge was removed. The stress developed during the distraction of the open wedge was not considered. Finally, the residual stress between the bone and plate developed during the screw inserting was not considered.

## Conclusion

LHFs drastically reduce HTO stability; hence, a fixation, in addition to the original medial TomoFix plate, is required to restore HTO stability in LHFs. Therefore, bilateral plating is a recommended fixation strategy to restore HTO stability in LHFs. Furthermore, the locking screw at the proximal combi hole (D-hole) is strongly recommended to reduce the stress on the TomoFix plate, thus reducing the risk of plate breakage in the future during postsurgery rehabilitation programs.

## Data Availability

All the data will be available upon motivated request to the corresponding authors of the present paper.

## Abbreviations

BP: Bilateral plating
BPWD: Bilateral plating without the locking screw at the proximal combi hole
D-Hole: Combi hole
FE: Finite element
HTO: High tibial osteotomy
KOA: Knee osteoarthritis
LHF: Lateral hinge fracture
MP: Medial plating
T1: Type 1
T2: Type 2
T3: Type 3
